# Association of EPA and DHA with age-related macular degeneration: a cross-sectional study from NHANES

**DOI:** 10.3389/fmed.2024.1440479

**Published:** 2024-09-04

**Authors:** Kewei Li, Jialing Liu, Xuhui Li, Xiaozhu Liu, Pengcheng Hu, Ming He

**Affiliations:** ^1^Department of Ophthalmology, the First Affiliated Hospital of Chongqing Medical University, Chongqing, China; ^2^Department of Phase I Clinical Trial Center, the Second Affiliated Hospital of Chongqing Medical University, Chongqing, China; ^3^Department of Ophthalmology, the Second Affiliated Hospital of Chongqing Medical University, Chongqing, China; ^4^Emergency and Critical Care Medical Center, Beijing Shijitan Hospital, Capital Medical University, Beijing, China

**Keywords:** age-related macular degeneration, eicosapentaenoic acid (EPA), docosahexaenoic acid (DHA), dose-response, NHANES

## Abstract

**Purpose:**

This cross-sectional study conducted in the general US population investigated the association between dietary intake of eicosapentaenoic acid (EPA) and docosahexaenoic acid (DHA) and the prevalence of AMD.

**Methods:**

Data from the National Health and Nutrition Examination Survey (NHANES) were utilized, including 4,842 participants aged 40 years and older. Dietary EPA and DHA intake data were collected through two 24-h dietary recall interviews and adjusted for weight. AMD was determined by a standardized grading system based on the presence of key features of AMD in color photographs of the macula. Multivariate logistic regression and restricted cubic spline models evaluated the associations between dietary EPA and DHA intake and AMD. Subgroup analysis and interaction analysis explored the influence of covariates.

**Results:**

A total of 4,842 participants were included. In the multivariate-adjusted model 2, the odds ratios (ORs) with 95% confidence intervals (CIs) for AMD were 0.86 (0.75, 0.99) and 0.88 (0.80, 0.97) per unit increase in dietary EPA and DHA intake, respectively. Interaction testing revealed significant effect modification by age, education, and BMI on the EPA-AMD association, indicating these factors significantly impacted this inverse relationship (*p*-interaction < 0.05). Similarly, age, education, BMI, and cataract surgery history modified the inverse DHA-AMD association (*p*-interaction < 0.05). Dose-response analyses demonstrated a negative correlation between dietary EPA and DHA intake with AMD prevalence (*p*-nonlinearity = 0.184 and 0.548, respectively).

**Conclusion:**

Our findings suggested that higher dietary EPA and DHA intake could be associated with lower AMD risk in older US adults. Age, education level, BMI, and history of cataract surgery may influence this inverse association.

## 1 Introduction

Age-related macular degeneration (AMD) is a disease that damages the macular region of the retina. It can be neovascular (also known as wet or exudative) or non-neovascular (known as atrophic, dry, or non-exudative). Both result in the loss of central visual acuity, leading to severe and permanent visual impairment and irreversible blindness (1, 2). In 2020, AMD ranked as the fourth leading cause of blindness worldwide and the third most common cause of moderate and severe vision impairment (MSVI). Moreover, AMD represents the primary cause of severe vision loss among individuals over 50 years of age in high-income nations (3). Age-related macular degeneration is estimated to be present in 8.69% of the world’s population. The projected number of people with this disease in 2020 is 196 million; This prevalence is expected to increase to 288 million in 2040 (4).

N-3 polyunsaturated fatty acids are a class of essential polyunsaturated fatty acids that are crucial for human health. They include alpha-linolenic acid (ALA), eicosapentaenoic acid (EPA), and docosahexaenoic acid (DHA). ALA, the principal dietary omega-3 fatty acid, serves as a precursor to EPA, which in turn is the precursor for DHA. Docosahexaenoic acid (DHA), a long-chain omega-3 (n-3) polyunsaturated fatty acid, is a predominant lipid constituent of the membranes of the outer segment of retinal photoreceptors, and possesses anti-inflammatory and anti-angiogenic properties (5). It affects both the survival and development of neurons and retinal vascular cells (6, 7). EPA inhibits eicosanoids derived from arachidonic acid (AA), which have been implicated in retinal neovascular abnormalities, increased vascular permeability, and inflammatory processes (8, 9). EPA also enhances cellular antioxidant capacity by optimizing mitochondrial function and biogenesis, thus highlighting the potential of EPA supplementation to mitigate oxidative damage associated with chronic diseases (10). Studies in adult humans demonstrate that the conversion of α-Linolenic acid (ALA) to EPA and DHA is generally poor. Therefore, we need to eat foods that provide adequate amounts of EPA and DHA, including fish and fish oil products (11).

Clinical studies of the effects of omega-3 fatty acids on AMD have been inconsistent. Several studies showed that a higher intake of n-3 fatty acids was associated with a lower risk of AMD progression (12, 13). For example, Agron et al. (14) found that higher dietary intake of long-chain polyunsaturated fatty acids, such as DHA and EPA, was associated with a reduced risk of advanced age-related macular degeneration (AMD), particularly for the geographic atrophy (GA) subtype. However, other studies have different results. A prospective cohort study found that for patients with unilateral exudative AMD, 3 years of oral DHA-enriched supplementation had the same effect on the incidence of choroidal neovascularization (CNV) in the second eye as placebo (15). The Age-Related Eye Disease Study 2 (AREDS2) Randomized Clinical Trial showed that DHA+EPA did not further reduce the risk of progression to advanced AMD (16). A Cochrane review showed that omega-3 fatty acids supplementation in people with AMD for periods of up to 5 years did not reduce the risk of progression to advanced AMD. Therefore, currently available evidence does not support increasing dietary intake of omega-3 fatty acids for the explicit purpose of preventing or slowing the progression of AMD (17).

Because of the inconsistency of the above results, we conducted this cross-sectional study using data from NHANES 2005–2008 to explore the associations between EPA and DHA intake with the risk of AMD, and to explore the corresponding dose-response relationships.

## 2 Materials and methods

### 2.1 Study design and data source

We examined this association utilizing data from the National Health and Nutrition Examination Survey (NHANES) 2005–2008 for the US population. NHANES employs a complex, multistage probability sampling design to target a nationally representative sample of non-institutionalized civilians, implemented by the National Center for Health Statistics (NCHS). Appropriate weighting schemes were utilized throughout the analysis to provide optimal prevalence estimates for the US population. Data collection was performed via in-person interviews, telephone interviews, mobile medical examinations, and laboratory testing. The National Center for Health Statistics Ethics Review Board approved the study protocol (2005–2006), and informed consent was obtained from all participants for ongoing research through at-home interviews and examinations.

**FIGURE 1:**
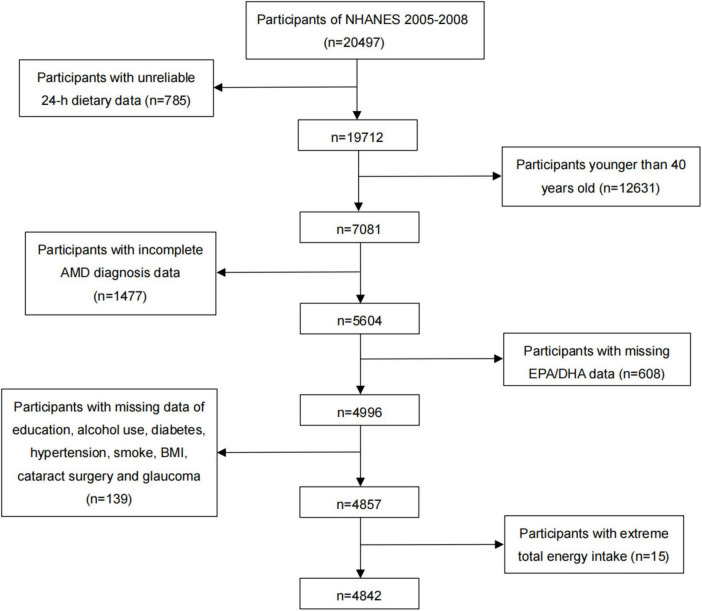
Flow chart for the selection of eligible participants for this study.

In NHANES 2005–2008, there were a total of 20,497 individuals, and our analyses were limited to 7,081 individuals over 40 years of age that had reliable 24-h dietary data. Among them, the individuals without complete AMD diagnosis and EPA or DHA data (*n* = 2,085), and with missing data on education, alcohol use, diabetes, hypertension, smoking, BMI, cataract surgery, and glaucoma (*n* = 139) were also excluded. Additionally, individuals with extreme total dietary energy intake were excluded from the study (males: < 500 or > 8,000 kcal/day, females: < 500 or > 5,000 kcal/day) (*n* = 15). Finally, we included a total of 4,842 participants in this cross-sectional study (Figure 1). We included the worse eye of each patient in our analysis.

### 2.2 Definition of AMD and ophthalmic examination

The main outcome was the presence of AMD diagnosed by NHANES on fundus photography. Two 45° non-mydriatic digital retinal images of each eye were obtained using fundus photography from an ophthalmic digital imaging system in participants 40 years and older. The first image was centered on the macula and the second on the optic nerve. The classification of age-related macular degeneration (AMD) was determined based on the condition of the worse eye. The grading team included nine experienced graders. The graders viewed each retinal image with a high-resolution monitor, using the EyeQLite image processing software and database, and referenced the written protocol and the digital photographic standards to evaluate retinal abnormalities (18). Early AMD was characterized by the presence or absence of drusen and/or pigmentary abnormalities, while late AMD was indicated by signs of exudative AMD and/or geographic atrophy (18). All images were graded by at least two individuals; When the first two inspectors failed to reach a consensus regarding a diagnosis, a third assessor examined the eye photographs.

### 2.3 Dietary intake of EPA and DHA

Dietary intake of DHA and EPA was assessed utilizing two 24-h dietary recalls. The primary recall was performed in person at the Mobile Examination Center (MEC), while the secondary recall was conducted via telephone interview 3 to 10 days subsequently. Participants reported foods and beverages consumed in the 24 h preceding the interview (midnight to midnight), and their reported food intake was used to calculate energy and nutrients. Comprehensive dietary interview procedures are accessible per NHANES dietary interviewer guideline manuals (19). DHA and EPA intake were estimated based on a detailed 2-day dietary recall from the participants, which was then analyzed using the United States Department of Agriculture’s Food and Nutrient Database for Dietary Studies, 3.0 (FNDDS 3.0). This database facilitated the calculation of nutrient intake from various food items consumed by the subjects, allowing for an accurate estimation of their DHA and EPA consumption (20). These intake values were then normalized by body weight (mg/kg/day) and partitioned into quartiles.

### 2.4 Covariates

Age, gender, race, and educational level were collected in demographic data. The poverty income ratio (PIR) was calculated by dividing the total income of a household by the poverty threshold for that household size, multiplied by the square root of the number of people in the household. A ratio of 1.0 or higher indicates that the household’s income is at or above the poverty level, while a ratio below 1.0 signifies that the household’s income is below the poverty threshold. Smoking status was obtained from cigarette use in the questionnaire and classified into 3 levels. The 3 levels were defined as never (smoked < 100 cigarettes in a lifetime), former (smoked ≥ 100 cigarettes in life and smoke not at all now), and now (have smoked ≥ 100 cigarettes in a lifetime and currently smoke). Alcohol consumption was categorized into five levels based on questionnaire data: never drinking (< 12 lifetime alcoholic drinks), former drinking (≥ 12 drinks/year but none in the past year), heavy drinking (≥ 4 and ≥ 3 drinks/day for men and women or binge drinking > 4 days/month), moderate drinking (≥ 3 and ≥ 2 drinks/day for men and women or binge drinking 1–4 days/month), and mild drinking (below moderate drinking criteria). Body mass index (BMI) was acquired from body measures in the examination data, which were calculated as weight in kilograms divided by height in meters squared. Hypertension was defined by any of the following criteria: (1) self-reported physician diagnosis on the questionnaire; (2) use of antihypertensive medication; or (3) systolic blood pressure ≥ 140 mmHg or diastolic blood pressure ≥ 90 mmHg. Hyperlipidemia was defined as meeting one or more of the following: (1) serum total cholesterol ≥ 200 mg/dL [5.18 mmol/L]; (2) triglycerides ≥ 150 mg/dL; (3) HDL cholesterol < 40 mg/dL [1.04 mmol/L] in men and < 50 mg/dL [1.30 mmol/L] in women; (4) LDL cholesterol ≥ 130 mg/dL [3.37 mmol/L]; or (5) use of cholesterol-lowering medication. Diabetes was defined by the presence of one or more of the following: (1) fasting plasma glucose ≥ 7.0 mmol/L; (2) random or 2-h OGTT plasma glucose ≥ 11.1 mmol/L; (3) glycated hemoglobin > 6.5%; (4) use of anti-diabetic medication; or (5) self-reported physician diagnosis. Cardiovascular diseases (CVD) were defined as meeting the following conditions:(1) coronary heart disease; (2) congestive heart failure; (3) heart attack; (4) stroke; (5) angina. Comorbid eye conditions include a self-reported history of cataract operation and glaucoma.

### 2.5 Statistical analysis

Per NHANES analytical guidelines, novel sample weights were derived by halving the original weights when amalgamating two cycles (NHANES 2005–2008) for this analysis. Crude weights were obtained from the two-day dietary sample weights. Continuous variables were conveyed as weighted mean ± standard error (SE). Categorical variables were conveyed as weighted percentages (95% confidence interval). The Student’s *t*-test for survey data and Rao-Scott chi-square test were utilized to juxtapose means of continuous variables and percentages of categorical variables between the AMD and non-AMD groups, respectively. Associations between dietary DHA and EPA and weighted odds ratios (95% confidence intervals) for AMD were calculated by multivariate logistic regression, which was adjusted by relevant variables. Model 1 was adjusted for age, sex, and race. Model 2 was adjusted for age, sex, race, education level, poverty income ratio, smoking status, alcohol intake, BMI, diabetes, hypertension, CVD, hyperlipidemia, cataract operation, and history of glaucoma. To probe the EPA/DHA-AMD relationship across subpopulations, subgroup analysis was undertaken. For each categorical variable, we conducted separate analyses to explore the relationship between EPA or DHA intake and AMD. For the continuous variable of age, we divided it into two subgroups for analysis: 40–64 years old and 65 years old or above. This categorization was based on the distribution characteristics of our data and clinical relevance, facilitating the analysis and interpretation. Interaction analyses were performed by including multiplicative interaction terms in our statistical models. We assessed whether the effect of EPA or DHA intake on AMD risk was modified by other variables. Specifically, we tested for significant interactions by including cross-product terms of EPA or DHA with other covariates in the model and evaluated the significance of the coefficients associated with these terms. We considered interactions to be statistically significant if the *p* < 0.05. Additionally, dose-response relationships were scrutinized employing logistic regression modeling with restricted cubic spline (RCS) in the definitive analysis model. Nonlinear *p*-values were derived. A *p*-value for nonlinearity below 0.05 signifies the presence of a statistically significant nonlinear association in the logistic regression model with RCS. All statistical analyses were performed using the R language. A two-tailed *P* < 0.05 was considered statistically significant.

## 3 Results

Demographics of the study participants, encompassing 383 subjects with AMD and 4,459 without AMD, are depicted in Table 1. The prevalence of AMD was 7.91% (383/4,842). The mean age was 68.86 ± 0.94 and 55.91 ± 0.36 years in each group, respectively. There were substantial variations between the groups in terms of age, race, poverty income ratio (PIR), Smoking, alcohol intake, diabetes, hypertension, CVD, Hyperlipidemia, Cataract operation, and History of glaucoma (*P* < 0.05). Furthermore, DHA and EPA intake in non-AMD participants was significantly higher than in AMD participants (*P* < 0.05).

**TABLE 1:** Characteristics of the participants: a cross-sectional study using data from NHANES 2005–2008 (*N* = 4,842).

Characteristic	All participants (*N* = 4,842)	Without AMD (*n* = 4,459)	With AMD (*n* = 383)	*P*-value
Mean age (± SE), year	56.77 ± 0.39	55.91 ± 0.36	68.86 ± 0.94	< 0.001
Age group				< 0.001
40–64	73.79 (65.77, 81.82)	76.81 (74.74, 78.88)	31.27 (24.80, 37.75)	
≥ 65	26.21 (22.63, 29.78)	23.19 (21.12, 25.26)	68.73 (62.25, 75.20)	
Sex				0.532
Female	54.31 (48.75, 59.88)	54.17 (52.50, 55.84)	56.30 (50.15, 62.45)	
Male	45.69 (40.26, 51.11)	45.83 (44.16, 47.50)	43.70 (37.55, 49.85)	
Race/ethnicity				< 0.001
Non-Hispanic White	78.60 (67.32, 89.88)	77.86 (73.83, 81.88)	89.03 (85.87, 92.19)	
Non-Hispanic Black	9.38 (7.64, 11.12)	9.78 (7.29, 12.27)	3.73 (2.39, 5.07)	
Mexican American	5.14 (3.96, 6.31)	5.25 (3.91, 6.58)	3.55 (2.00, 5.11)	
Other Race	6.89 (5.06, 8.71)	7.11 (5.28, 8.94)	3.69 (1.12, 6.25)	
Education				0.239
Less than high school	16.42 (13.85, 18.99)	16.10 (14.05, 18.16)	20.90 (14.11, 27.69)	
High school graduate/GED	26.65 (22.98, 30.32)	26.63 (24.30, 28.96)	27.00 (20.92, 33.08)	
Some colleges or above	56.93 (49.83, 64.02)	57.27 (53.51, 61.03)	52.10 (44.46, 59.74)	
Poverty income ratio (PIR)				< 0.001
< 1	8.33 (7.06, 9.61)	8.36 (7.15, 9.58)	7.91 (4.79, 11.03)	
1–3	32.01 (27.25, 36.78)	31.07 (27.40, 34.74)	45.31 (38.50, 52.13)	
> 3	54.18 (46.44, 61.92)	55.32 (50.93, 59.71)	38.03 (30.89, 45.17)	
Unknown	5.47 (4.26, 6.68)	5.24 (4.07, 6.41)	8.75 (4.65, 12.84)	
Smoking status				< 0.001
Never	48.32 (42.32, 54.32)	48.93 (46.37, 51.48)	39.80 (33.95, 45.65)	
Former	31.39 (27.86, 34.91)	30.50 (28.05, 32.95)	43.88 (37.84, 49.93)	
Now	20.29 (17.00, 23.58)	20.57 (18.28, 22.87)	16.32 (11.02, 21.62)	
Alcohol intake				0.047
Never	10.77 (9.15, 12.38)	10.40 (8.66, 12.14)	15.90 (12.16, 19.63)	
Former	21.93 (18.94, 24.91)	21.68 (19.40, 23.95)	25.47 (21.14, 29.80)	
Mild	40.07 (34.78, 45.37)	40.33 (37.73, 42.93)	36.47 (29.08, 43.85)	
Moderate	15.24 (13.01, 17.48)	15.37 (13.95, 16.79)	13.42 (9.40, 17.44)	
Heavy	11.99 (10.06, 13.92)	12.22 (11.08, 13.37)	8.74 (4.05, 13.43)	
BMI (kg/m^2^) (%)				0.191
< 18.5	1.17 (0.75, 1.60)	1.22 (0.79, 1.65)	0.48 (−0.08, 1.04)	
18.5–30	62.26 (55.25, 69.27)	61.96 (59.27, 64.65)	66.50 (60.30, 72.70)	
≥ 30	36.57 (31.89, 41.24)	36.82 (34.15, 39.49)	33.02 (26.86, 39.19)	
Diabetes				0.047
No	83.71 (74.32, 93.10)	84.06 (82.24, 85.88)	78.85 (72.90, 84.80)	
Yes	16.29 (13.98, 18.59)	15.94 (14.12, 17.76)	21.15 (15.20, 27.10)	
Hypertension				< 0.001
No	50.32 (44.05, 56.60)	51.50 (48.74, 54.25)	33.78 (26.74, 40.81)	
Yes	49.68 (44.07, 55.29)	48.50 (45.75, 51.26)	66.22 (59.19, 73.26)	
CVD				< 0.001
No	88.24 (78.87, 97.61)	89.28 (88.08, 90.48)	73.66 (67.83, 79.49)	
Yes	11.76 (9.90, 13.61)	10.72 (9.52, 11.92)	26.34 (20.51, 32.17)	
Hyperlipidemia				0.018
No	19.26 (17.58, 20.94)	19.67 (17.98, 21.36)	13.48 (9.48, 17.47)	
Yes	80.74 (71.18, 90.30)	80.33 (78.64, 82.02)	86.52 (82.53, 90.52)	
Cataract operation				< 0.001
No	90.65 (81.07, 100.22)	92.28 (91.46, 93.10)	67.59 (61.72, 73.45)	
Yes	9.35 (7.93, 10.78)	7.72 (6.90, 8.54)	32.41 (26.55, 38.28)	
History of glaucoma				< 0.001
No	95.05 (85.02, 105.08)	95.54 (94.71, 96.38)	88.07 (83.90, 92.25)	
Yes	4.95 (3.95, 5.95)	4.46 (3.62, 5.29)	11.93 (7.75, 16.10)	
Eicosapentaenoic (mg/kg/day)	0.67 ± 0.06	0.69 ± 0.06	0.37 ± 0.05	< 0.001
Docosahexaenoic (mg/kg/day)	1.21 ± 0.10	1.24 ± 0.11	0.70 ± 0.07	< 0.001

Continuous variables were expressed as weighted mean ± standard error (SE). Categorical variables were expressed as weighted percentages (95% confidence interval). *P*-values were weighted by dietary two-day sample weight. AMD, age-related macular degeneration; CVD, cardiovascular disease; BMI, body mass index.

Weighted odds ratios (ORs) with 95% confidence intervals (95% CIs) of AMD based on EPA and DHA are shown in Tables 2, 3, respectively. In the unadjusted model, both were significantly associated with AMD (OR: 0.84, 95% CI: 0.76, 0.94; OR: 0.85, 95% CI: 0.78, 0.93) (*p* < 0.05), respectively. In Model 1, after adjusting for age, gender, and race, EPA and DHA intakes were still inversely associated with the risk of AMD. After further adjusting for education level, poverty income ratio, smoking status, alcohol intake, BMI, diabetes, hypertension, CVD, hyperlipidemia, cataract operation, and history of glaucoma in Model 2, the ORs with 95% CIs of AMD were 0.86 (0.75, 0.99) and 0.88 (0.80, 0.97) for EPA and DHA, respectively. The trend analysis for EPA intake demonstrates a significant protective effect against AMD, with a statistically significant trend (*P* for trend < 0.05). In contrast, for DHA intake, the trend analysis in model 2 does not show a significant relationship with the risk of AMD. This lack of significance suggests that the protective effect of DHA may not be as consistent or as strong as that of EPA.

**TABLE 2:** Weighted odds ratios (95% confidence intervals) of AMD with adjusted dietary eicosapentaenoic intake (mg/Kg/day).

	Crude	Model 1	Model 2
Eicosapentaenoic (mg/kg/day)	0.84 (0.76, 0.94)	0.85 (0.76, 0.96)	0.86 (0.75, 0.99)
**Quintiles of eicosapentaenoic**			
Q1 (*N* = 1,203) (0–0.034 mg/Kg/day)	Ref	Ref	Ref
Q2 (*N* = 1,223) (0.034–0.099 mg/Kg/day)	0.92 (0.58, 1.45)	0.87 (0.55, 1.38)	0.91 (0.51, 1.63)
Q3 (*N* = 1,206) (0.099–0.318 mg/Kg/day)	0.66 (0.43, 1.00)	0.76 (0.49, 1.17)	0.79 (0.44, 1.43)
Q4 (*N* = 1,210) (0.318–22.991 mg/Kg/day)	0.55 (0.38, 0.81)	0.59 (0.40, 0.87)	0.62 (0.38, 1.00)
*P* for trend	0.002	0.008	0.035

Crude: non-adjusted model; Model 1 adjusted for age, sex, race; Model 2 adjusted for age, sex, race, education level, poverty income ratio, smoking status, alcohol intake, BMI, diabetes, hypertension, CVD, hyperlipidemia, cataract operation, and history of glaucoma.

**TABLE 3:** Weighted odds ratios (95% confidence intervals) of AMD with adjusted dietary docosahexaenoic intake (mg/Kg/day).

	Crude	Model 1	Model 2
Docosahexaenoic (mg/kg/day)	0.85 (0.78, 0.93)	0.87 (0.80, 0.95)	0.88 (0.80, 0.97)
**Quintiles of docosahexaenoic**			
Q1 (*N* = 1,212) (0–0.166 mg/Kg/day)	Ref	Ref	Ref
Q2 (*N* = 1,205) (0.166–0.399 mg/Kg/day)	0.84 (0.51, 1.38)	0.91 (0.54, 1.53)	0.91 (0.44, 1.85)
Q3 (*N* = 1,214) (0.399–0.993 mg/Kg/day)	0.68 (0.40, 1.14)	0.83 (0.47, 1.48)	0.86 (0.40, 1.84)
Q4 (*N* = 1,211) (0.993, 44.629 mg/Kg/day)	0.53 (0.35, 0.79)	0.63 (0.41, 0.99)	0.65 (0.36, 1.18)
*P* for trend	0.006	0.078	0.142

Crude: non-adjusted model; Model 1 adjusted for age, sex, and race; Model 2 adjusted for age, sex, race, education level, poverty income ratio, smoking status, alcohol intake, BMI, diabetes, hypertension, CVD, hyperlipidemia, cataract operation, and history of glaucoma.

The results of our subgroup analysis revealed that there were inconsistent relationships between dietary EPA and DHA with AMD (Tables 4, 5). Regarding the relationship between dietary EPA and AMD, stratified subgroups showing statistically significant associations included participants aged 40–64 years, some college or above, BMI ≥ 30 (kg/m2), without diabetes, CVD, hyperlipidemia, cataract operation, and history of glaucoma connection. A significant association of dietary DHA with AMD was observed in participants aged 40–64, Female, Non-Hispanic White, some college or above, BMI ≥ 30 (kg/m2), former drinking, hyperlipidemia, without diabetes and CVD, without history of glaucoma and cataract operation (all *p* < 0.05). Interaction analysis uncovered significant disparities based on age, education level, and BMI regarding the association between dietary EPA and AMD, indicating these variables substantially impacted this inverse relationship (*p*-interaction < 0.05). Additionally, age, education status, BMI, and cataract surgery history modulated the negative correlation between dietary DHA and AMD (*p*-interaction < 0.05).

**TABLE 4:** Subgroup analysis and interaction test for the association between dietary eicosapentaenoic intake (mg/Kg/day) and AMD.

Model 2	OR 95% CI	*p*	*p* for interaction
Age group			0.005
40–64	0.53 (0.29, 0.97)	0.041	
≥ 65	0.95 (0.81, 1.10)	0.431	
Sex			0.557
Female	0.83 (0.69, 1.00)	0.052	
Male	0.89 (0.73, 1.08)	0.194	
Race/ethnicity			0.210
Non-Hispanic White	0.87 (0.75, 1.00)	0.053	
Non-Hispanic Black	0.91 (0.52, 1.57)	0.675	
Mexican American	0.95 (0.77, 1.19)	0.735	
Other Race	0.30 (0.04, 2.11)	0.144	
Education			0.018
Less than high school	0.85 (0.63, 1.13)	0.218	
High school graduate/GED	1.00 (0.82, 1.22)	1.000	
Some colleges or above	0.72 (0.55, 0.94)	0.021	
Poverty income ratio (PIR)			0.372
< 1	1.19 (0.87, 1.63)	0.235	
1–3	0.87 (0.69, 1.08)	0.178	
> 3	0.83 (0.67, 1.02)	0.073	
Unknown	0.62 (0.37, 1.06)	0.073	
Smoking status			0.678
Never	0.92 (0.73, 1.14)	0.386	
Former	0.83 (0.67, 1.03)	0.076	
Now	0.78 (0.41, 1.47)	0.390	
Alcohol intake			0.749
Never	0.92 (0.68, 1.23)	0.531	
Former	0.70 (0.47, 1.03)	0.068	
Mild	0.91 (0.75, 1.11)	0.307	
Moderate	0.90 (0.71, 1.15)	0.353	
Heavy	0.72 (0.41, 1.27)	0.230	
BMI (kg/m^2^) (%)			0.009
18.5–30	0.92 (0.81, 1.05)	0.168	
≥ 30	0.52 (0.33, 0.82)	0.011	
Diabetes			0.541
No	0.84 (0.74, 0.96)	0.019	
Yes	0.96 (0.65, 1.42)	0.816	
Hypertension			0.105
No	0.91 (0.78, 1.08)	0.231	
Yes	0.78 (0.58, 1.03)	0.074	
CVD			0.091
No	0.79 (0.65, 0.98)	0.033	
Yes	1.02 (0.80, 1.29)	0.880	
Hyperlipidemia			0.141
No	0.68 (0.48, 0.97)	0.037	
Yes	0.88 (0.77, 1.00)	0.052	
Cataract operation			0.056
No	0.82 (0.67, 0.99)	0.043	
Yes	1.04 (0.85, 1.28)	0.651	
History of glaucoma			0.453
No	0.86 (0.75, 0.99)	0.043	
Yes	0.75 (0.43, 1.32)	0.261	

Model 2 was a fully adjusted model, and the stratified variables in the subgroup analysis were not adjusted.

**TABLE 5:** Subgroup analysis and interaction test for the association between dietary docosahexaenoic intake (mg/Kg/day) and AMD.

Model 2	OR 95% CI	*p*	*p* for interaction
Age group			0.032
40–64	0.71 (0.51, 0.98)	0.042	
≥ 65	0.94 (0.83, 1.06)	0.235	
Sex			0.448
Female	0.85 (0.74, 0.98)	0.030	
Male	0.91 (0.79, 1.04)	0.145	
Race/ethnicity			0.385
Non-Hispanic White	0.88 (0.79, 0.98)	0.026	
Non-Hispanic Black	0.92 (0.64, 1.31)	0.568	
Mexican American	0.96 (0.81, 1.13)	0.678	
Other Race	0.61 (0.30, 1.27)	0.123	
Education			0.026
Less than high school	0.86 (0.68, 1.07)	0.152	
High school graduate/GED	0.97 (0.84, 1.13)	0.695	
Some college or above	0.79 (0.68, 0.92)	0.008	
Poverty income ratio (PIR)			0.655
< 1	1.11 (0.87, 1.42)	0.354	
1–3	0.86 (0.72, 1.03)	0.090	
> 3	0.87 (0.75, 1.01)	0.062	
Unknown	0.83 (0.62, 1.11)	0.170	
Smoking status			0.557
Never	0.92 (0.79, 1.07)	0.259	
Former	0.86 (0.73, 1.02)	0.075	
Now	0.76 (0.46, 1.25)	0.240	
Alcohol intake			0.808
Never	0.91 (0.74, 1.12)	0.353	
Former	0.72 (0.56, 0.93)	0.018	
Mild	0.92 (0.80, 1.05)	0.202	
Moderate	0.92 (0.76, 1.13)	0.399	
Heavy	0.84 (0.64, 1.11)	0.193	
BMI (kg/m^2^) (%)			0.042
18.5–30	0.92 (0.84, 1.02)	0.088	
≥ 30	0.66 (0.45, 0.95)	0.030	
Diabetes			0.565
No	0.86 (0.78, 0.95)	0.010	
Yes	0.95 (0.70, 1.30)	0.726	
Hypertension			0.167
No	0.90 (0.80, 1.02)	0.092	
Yes	0.83 (0.68, 1.00)	0.052	
CVD			0.119
No	0.83 (0.72, 0.96)	0.022	
Yes	1.00 (0.83, 1.20)	0.986	
Hyperlipidemia			0.401
No	0.76 (0.58, 1.00)	0.050	
Yes	0.89 (0.80, 0.98)	0.026	
Cataract operation			0.030
No	0.83 (0.72, 0.96)	0.021	
Yes	1.04 (0.89, 1.21)	0.561	
History of glaucoma			0.570
No	0.88 (0.79, 0.98)	0.023	
Yes	0.83 (0.56, 1.24)	0.300	

Model 2 was a fully adjusted model, and the stratified variables in the subgroup analysis were not adjusted.

**FIGURE 2:**
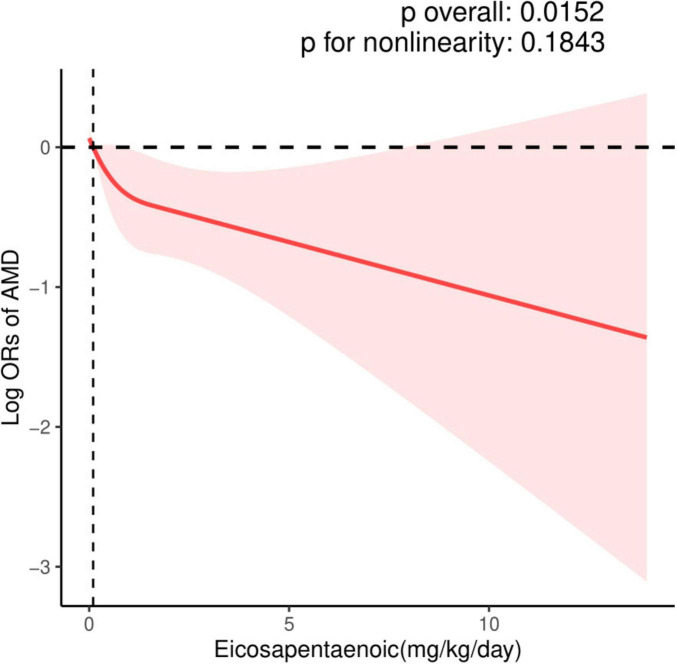
Restricted cubic spline (RCS) plot of the association between dietary EPA intake and the presence of AMD.

**FIGURE 3:**
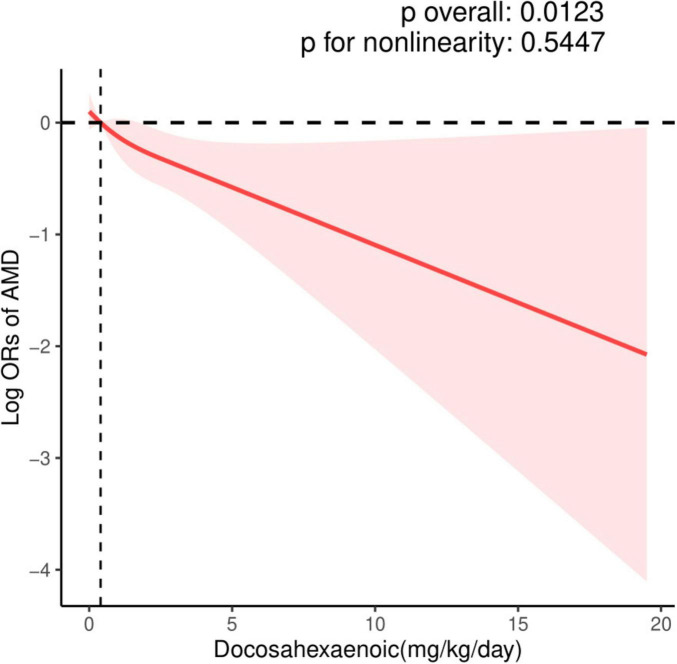
Restricted cubic spline (RCS) plot of the association between dietary DHA intake and the presence of AMD.

The dose-response relationship between EPA and DHA intake with AMD prevalence is exhibited in Figures 2, 3. A negative correlation association was evident between EPA intake and AMD prevalence (nonlinearity *p* = 0.184). AMD prevalence declined with rising EPA intake; no discernible threshold or inflection point was apparent. Moreover, the DHA-AMD prevalence dose-response relationship was similar (nonlinearity *p* = 0.548), implying that AMD risk decreased with escalating dietary DHA.

## 4 Discussion

In this cross-sectional study of nationally representative US adults, we investigated the associations between dietary DHA and EPA with the risk of AMD among older adults in the United States. we found that dietary intakes of DHA and EPA were associated with a lower prevalence rate of AMD. We also found that age, education, BMI, and history of cataract operation may influence this negative association. The dose-response relationship revealed that there were negative associations of dietary DHA and EPA with the prevalence of AMD.

Our results support previous studies that have found an inverse correlation between DHA and EPA and AMD risk. A multicenter, prospective cohort study indicated a significant correlation between elevated DHA levels as well as increased combined DHA+EPA levels and decreased risk of early AMD. However, EPA levels alone did not exhibit a significant association with AMD (21). A randomized controlled study found that serum EPA was significantly associated with a lower risk of neovascular AMD (OR = 0.41; 95% CI 0.22–0.77; *p* = 0.005). Analysis of red blood cell membranes (RBCM) revealed that EPA and EPA+DHA were significantly associated with a lower risk of neovascular AMD (OR = 0.25; 95% CI 0.13–0.47; *p* < 0.0001 and OR = 0.52; 95% CI 0.29–0.94; *p* = 0.03, respectively) (22). Augood et al. (12) found that eating oily fish at least once a week compared to less than once a week was associated with a halving of the odds of NV-AMD (OR 0.47; 95% CI 0.33–0.68; *p* = 0.002) (12). Reynolds et al. (23) also indicated that an increase in self-reported dietary intake of omega-3 fatty acids (DHA and EPA) was significantly associated with a reduced risk of AMD progression (23).

In contrast, the results of several studies were not entirely consistent. In a randomized, double-blind, and comparative study, the patients were randomized to be allocated either 840 mg/day DHA and 270 mg/day EPA from fish oil capsules or placebo (olive oil capsules) for a 3-year duration. Time to onset and incidence of CNV in the study eye exhibited no significant differences between the DHA supplementation group compared to placebo (15). The Age-Related Eye Disease Study 2 (AREDS2) found that DHA+EPA did not further reduce the risk of progression to advanced AMD (24). A review also found that omega-3 fatty acids supplementation in people with AMD for periods of up to five years does not reduce the risk of progression to advanced AMD or the development of moderate to severe visual loss (17). However, there are several potential explanations for the negative result between omega-3 fatty acids supplementation and AMD. Firstly, the relatively low doses of DHA (350 or 840 mg) and EPA (650 or 270 mg) used in those trials may cause the results. A total of 1,027 patients were randomized to be allocated either approximately 1.6 g/day of long-chain omega-3 fatty acids (930 mg EPA and 660 mg DHA) or corn oil placebo as an adjunct to standard therapy. Following 2 years of follow-up, no significant differences were evident between the supplemented and control cohorts for the primary composite cardiovascular endpoint (25). However, a meta-regression analysis demonstrated that each 1 g/day increment in EPA+DHA intake conferred a 5.8% decrease in CVD risk. For myocardial infarction (MI) specifically, the risk reduction exhibited a dose-dependent relationship, with each supplemental 1 g/day of EPA+DHA conferring a significant 9% lowering of risk (25). Our study also found that the higher the dietary DHA and EPA, the lower the incidence of AMD, and it is a quantity-dependent relationship. Second, in AREDS2, participants were taking the AREDS1 formula; therefore, the effect of adding dietary DHA+EPA may have been masked by the base formula and an additional benefit could not be detected. Another additional explanation for the failure of omega-3 in these studies was that the follow-up time was short, and age is the most important influencing factor of AMD (2), so a long follow-up time can better observe the effect of DHA and EPA in the diet.

EPA and DHA are both long-chain omega-3 fatty acids. The body’s ability to synthesize them from the precursor ALA is not efficient, so their direct intake through the diet may be necessary to obtain health benefits (26). The retina is one of the tissues with the highest lipid content in the human body. The major omega-3 fatty acid, DHA, is a major structural component of the retinal photoreceptor membranes and the vascular tissue of the retina (8). DHA and EPA have been reported to have suppressive effects against oxidative stress and inflammation (27). However, the precise mechanisms underlying the association between EPA and DHA intake and reduced AMD risk remain incompletely elucidated. Nonetheless, various potential mechanisms have been proposed. EPA and DHA intake may attenuate the production of inflammatory cytokines and eicosanoids (8). DHA can also negatively modulate vascular cell adhesion molecule 1 (VCAM-1) expression induced by tumor necrosis factor α (TNF-α) via dampening of nuclear factor-κB (NF-κB) signaling and activator protein-1 (AP-1) activation, thereby exerting anti-inflammatory properties (28). EPA application substantially reduced the expression of intercellular adhesion molecule 1 (ICAM-1) and monocyte chemotactic protein 1 (MCP-1) in endothelial cells and the expression of VEGF and interleukin-6 (IL-6) in macrophages, effects which have anti-inflammatory and anti-angiogenic properties (29), and it is known that inflammatory effects play an important role in the occurrence and development of AMD (27). A key target in modulating lipid metabolism is the peroxisome proliferator-activated receptor (PPAR), which has been demonstrated to augment macrophage phagocytosis of apoptotic cells, promote M2 macrophage polarization, decrease matrix metalloproteinase (MMP) 2 and MMP9, and suppress choroidal neovascularization (CNV) (30, 31). Both DHA and EPA are ligands of PPAR isoforms (32). Previous studies also demonstrated that omega-3 (DHA and EPA) supplementation can decrease vitreal VEGF levels in patients with wet AMD (33), and significantly improve plasma antioxidant capacity (34).

In addition, we also found that age, education, BMI, and history of cataract operation can influence the prevalence of AMD, which had also been reported in previous studies (35–37). The intricate role of aging in the pathogenic mechanisms underlying AMD remains incompletely elucidated, potentially attributable to oxidative stress, amyloid beta accumulation, circadian rhythm dysfunction, metabolic aging, and cellular senescence impacting photoreceptors, microglia, RPE cells, Bruch’s membrane, and the choroid (36). A Korean study also suggested that increased education was a unique risk factor for AMD (38). The Age-Related Eye Disease Study (AREDS) found that greater body mass was associated with a higher risk of AMD (39). One possible explanation is that excessive caloric intake increased the risk of developing AMD, as it could have led to increased oxidative stress that damages the retina (40). One putative hypothesis for the heightened AMD prevalence following cataract surgery entails greater retinal exposure to blue light; photochemical reactions within the oxygen-replete outer retinal milieu engender cytotoxic reactive oxygen species (ROS). These ROS induce oxidative stress, a recognized contributor to AMD pathogenesis (41), while the exact mechanism remains to be further explored.

This study has several strengths, including a substantial nationally representative sample, comprehensive subgroup, interaction analyses, and augmented statistical power and result reliability conferred by the ample sample size. Furthermore, numerous potential confounders were adjusted for, encompassing health status along with socioeconomic and lifestyle variables. Additionally, dose-response relationships between dietary EPA/DHA intake and AMD risk were examined, reinforcing the conclusion that higher EPA/DHA consumption correlates with reduced AMD prevalence.

Certain limitations to this study must be acknowledged. Foremost, as with other observational studies, causal inferences regarding the relationship between DHA/EPA intake and AMD prevalence cannot be established. At the same time, the subgroup analyses are exploratory and hypothesis-generating. Regarding the positive covariates in the results, they need to be approached with caution and further research is required for validation. Second, we did not assess the associations of DHA and EPA levels in serum and red-blood-cell membranes (RBCM) with AMD, while RBCM EPA and EPA+DHA, as biomarkers of the dietary status of n-3 fatty acids, were strongly associated with AMD and can represent an objective biomarker (22). Third, dietary data was collected via 24-h dietary recalls, a survey method often hampered by inaccurate conscious or unconscious recording and underreporting, potentially subject to recall bias. Lastly, we were unable to obtain the dietary supplement intake of DHA and EPA in NHANES, which was an important source of DHA and EPA.

## 5 Conclusion

In conclusion, our investigation revealed a negative correlation between the prevalence of AMD and dietary EPA and DHA in the older adult population of the United States. These findings imply that dietary EPA and DHA intake may exhibit an inverse correlation with AMD risk among older adults in the US. Age, education level, BMI, and cataract surgery history appear to impact this negative association. Additional large-scale prospective cohort studies alongside further biological experiments are warranted to validate these results and elucidate the precise underlying mechanisms.

## Data Availability

Publicly available datasets were analyzed in this study. This data can be found here: https://www.cdc.gov/nchs/nhanes/index.htm.
